# Preparation of Lightweight Ceramsite from Solid Waste Using SiC as a Foaming Agent

**DOI:** 10.3390/ma15010325

**Published:** 2022-01-03

**Authors:** Shuo Shang, Haihong Fan, Yuxiang Li, Lin Li, Zhou Li

**Affiliations:** College of Materials Science and Engineering, Xi’an University of Architecture and Technology, Xi’an 710055, China; ss08152021@163.com (S.S.); yuxli2021@xauat.edu.cn (Y.L.); lilylee0823@163.com (L.L.); leezu1997@163.com (Z.L.)

**Keywords:** SiC, lightweight ceramsite, sintering expansion behavior, solid waste

## Abstract

SiC was chosen as the foaming agent, and river bottom silt, waste oil sludge, paint bucket slag, and fly ash were used as raw materials, to prepare lightweight ceramsite without adding any chemical additives. The effects of SiC dosing and sintering temperature on various properties of the ceramsite were studied, and the pore-forming mechanism of the lightweight ceramsite was clarified by thermal analysis and X-ray diffraction analysis. The results showed that the single ceramsite compressive strength, water absorption, bulk density, and porosity of ceramsite sintered at 1180 °C with 1.0% SiC were 2.15 MPa, 2.02%, 490 kg/m^3^, and 23.85%, respectively. The major mineralogical compositions were quartz, fayalite, and kyanite, with small amounts of albite-low from 1140 to 1190 °C. Furthermore, the concentration of all tested heavy metals from ceramsite was lower than the maximum allowable concentration of the leaching solution specified in the Chinese national standard (GB 5085.3-2007), which reveals that this solid waste ceramsite will not cause secondary environmental pollution. The prepared ceramsite, exhibiting lower bulk density, high water absorption and porosity, and effective solidification of deleterious elements, can be used to prepare green lightweight aggregate concrete. Importantly, preparation of solid waste ceramsite is an effective way to dispose of hazardous wastes.

## 1. Introduction

With the continuous global advancement of industrialization and urbanization, a variety of solid wastes harmful to the environment are increasingly being produced, such as fly ash, sludge, steel slag, etc. [1,2]. Most of these solid wastes contain large amounts of alkaline substances and heavy metals due to their production conditions, and can easily cause secondary pollution and threats to human health when handled and used [3,4]. To address the issue of the increasing amount of solid wastes and the need for environmentally-friendly uses, new strategies need to be urgently devised for the utilization of solid wastes. Ceramsite, which contains inorganic components such as Al_2_O_3_, SiO_2_, Fe_2_O_3_, FeO, CaO, MgO, Na_2_O, and K_2_O, has been used as a construction material, for example in cement mortars, concrete mixtures, bricks, as fine aggregate in mortars and ceramic materials, or as filter media in reactors for treating municipal/industrial wastewater/secondary effluent in wastewater treatment plants [5,6]. Traditional ceramsite is sintered with clay and shale, requiring high-quality clay and shale mines, which cause a certain degree of damage to the environment [7,8]. The large amount of silica-aluminous substances and other alkaline melts contained in solid waste are similar to the chemical components required for sintered ceramsite, and the preparation of ceramic pellets can be achieved by the reaction between different solid wastes. Solid waste ceramsite is a new type of environmental material, representing the development direction of ceramsite. In future, more than 95% of ceramsite will use solid waste as raw material for production [9].

The preparation of ceramsite from solid waste has been extensively researched. Mi, H et al. [10] prepared high-strength ceramsite from red mud, fly ash, and bentonite, which exhibited high compressive strength, low water absorption, and effective solidification. Moreover, the raw material weight ratio and sintering conditions, including sintering temperature, sintering time, preheating temperature, and preheating time were investigated. Ou, C. et al. [11] used two kinds of ceramsites with pH self-adjustment ability synthesized from lime mud and coal fly ash to efficiently control manganese pollution. The influence of different parameters like contact time, concentration of Mn^2+^, and pH on adsorption performance was examined. This study suggested ceramsites with pH self-adjustment ability have enormous potential in addressing the heavy metal pollution of water. Qin, J. et al. [6] used lime, coal fly ash, and various different additives (shale, perlite, diatomite, and sawdust) to prepare ceramsite, and analyzed the impacts of the sintering temperature and lime mud content on the physical properties of ceramsite. The results showed that anorthite was the major phase of ceramsites, except for ceramsite with 60 wt% lime mud. Wang, J. et al. [12] developed a novel ceramsite using sewer pipe sediment, riverbed sediment, urban water supply treatment sludge, and excess wastewater treatment plant sludge, and their new ceramsite could effectively remove Cu and Cd. In conclusion, research on the preparation of ceramsite from solid waste has mainly focused on the effects of different factors on the preparation process and performance of ceramsite, as well as the functional use of ceramic pellets, but the process of using solid waste to prepare lightweight ceramsite can still be improved.

Lightweight ceramsite is a light aggregate, with the advantages of low density, high strength, high water absorption, heat insulation, sound absorption, and noise reduction, etc. [13]. It can also be used as a water treatment filter, adsorbent, and permeable pavement material [14]. Due to its low density and high porosity, lightweight ceramsite in particular has excellent thermal insulation properties, and it is commonly used in the production of thermal concrete insulation and waterproofing materials [15,16]. Previous investigations have proved that clay and sewage sludge can successfully producing ultra-lightweight ceramsite (ULWC) and heavy metals of ULWC were properly stabilized [17,18]. However, it is still a great challenge to prepare lightweight ceramsite with good performance completely from solid wastes of different compositions or for its use at an industrial scale. The present study used SiC as the foaming agent to solve this problem through the following methods: (1) The properties and microscopic morphological characteristics of the ceramsite were used to determine the optimal amount of SiC as a foaming agent. (2) The effect of temperature on the properties of ceramsite was explored by X diffraction analysis and infrared spectroscopy, to determine the optimal firing temperature. (3) The expansion and pore formation mechanism of ceramsite was determined by thermal analysis, and used to illustrate the foaming process of SiC.

## 2. Materials and Methods

### 2.1. Materials

The raw materials used were river bottom silt (RBS), waste oil mud (OM), paint bucket slag (PBS), and fly ash (FA). River bottom silt is a yellowish clay block with close bonding. Most waste oil mud is black granular; a little is a hard large block. Paint bucket slag is grayish white and uneven in size, with a strong, pungent smell. Due to the high melting point of the above silt and the high iron content of the waste oil mud and paint bucket slag, fly ash was added to increase the SiO_2_ and Al_2_O_3_ contents. At this time, the chemical composition of the raw material can ensure that the liquid phase is sufficient and its viscosity is appropriate during sintering [19,20]. In order to improve the swelling effect, SiC powder (passed through a 45-μm sieve) was used as the foaming agent [21,22]. The compositions of the river bottom silt, waste oil mud, paint bucket slag, and fly ash were measured by X-ray fluorescence (XRF), and are presented in Table 1. It can be seen that the main components of river sludge are SiO_2_ (63.54%), the content of Fe_2_O_3_ in waste oil mud is as high as 52.49%, and the content of ZnO in paint bucket slag is 34.21%. The content of Fe_2_O_3_ is also large, at 18.66%. In addition, the four materials contained a number of flux elements, such as K, Mg, and Na, which were helpful for decreasing the calcination temperature.

**Figure 1:**
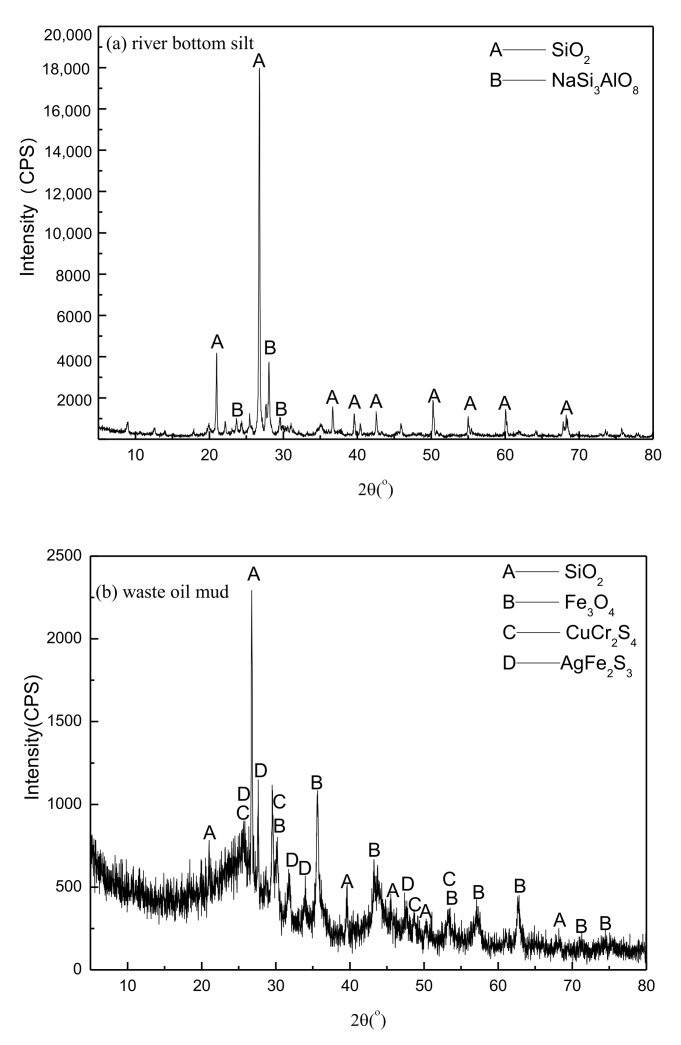

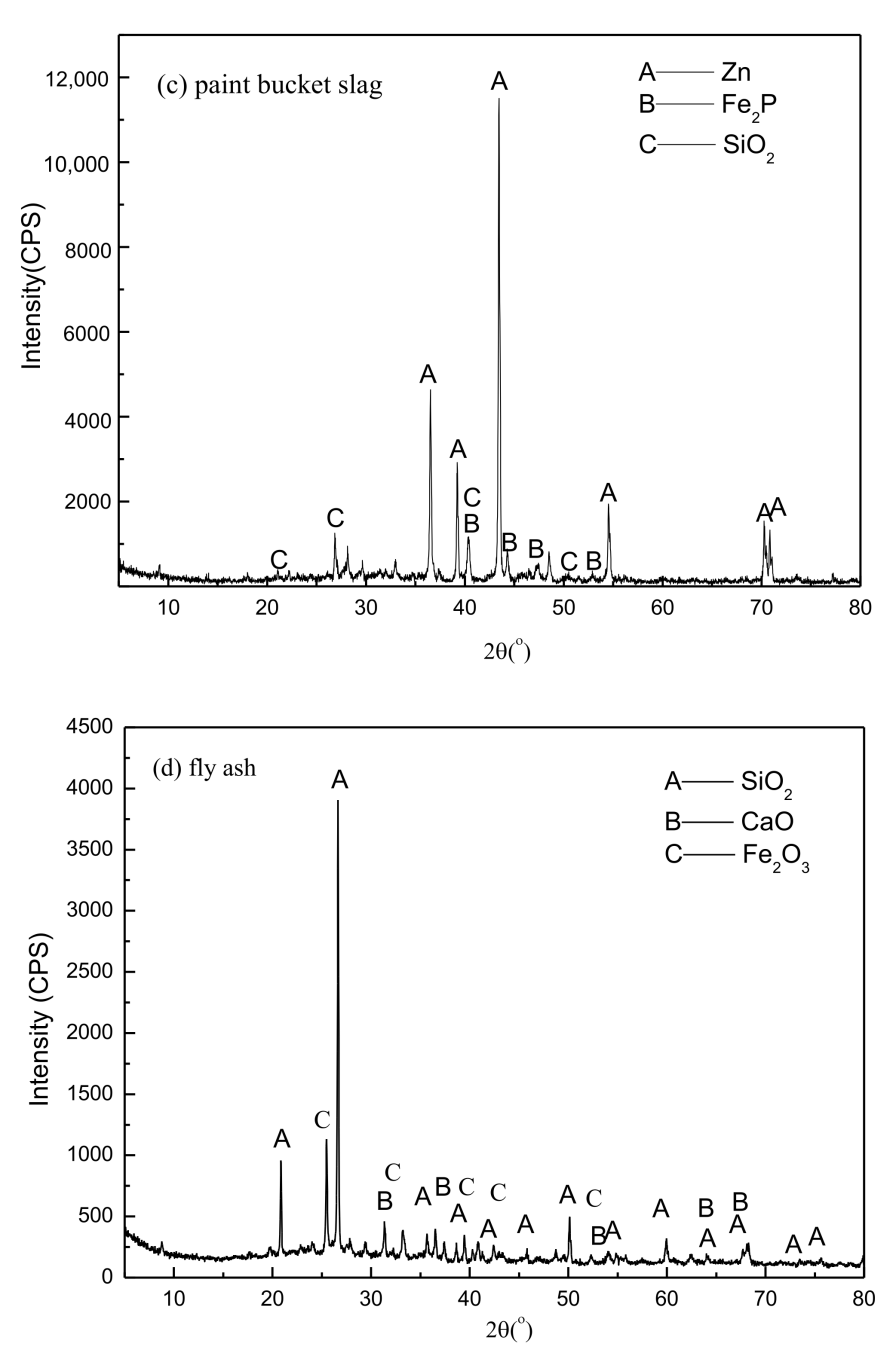
XRD spectra of river bottom silt (**a**)**,** waste oil mud (**b**), paint bucket slag (**c**), and fly ash (**d**).

The XRD spectra of river bottom silt, waste oil mud, paint bucket slag, and fly ash are shown in Figure 1. It revealed that the river bottom silt mainly contained silicon dioxide and albite, whereas the waste oil mud consisted of silicon dioxide and magnetite, and the main mineral phases in the paint residue were zinc metal, ferrophosphorus, and silicon dioxide. XRF and XRD analyses showed that the compositions of the raw materials were similar to that of natural clay. Therefore, the above materials can be used to prepare ceramsite.

### 2.2. Preparation of Ceramsite

According to Riley’s ternary phase diagram for the preparation of ceramsite, the optimum composition of the raw materials is 40–79% SiO_2_, 10–25% Al_2_O_3_, and 13–26% fluxes (CaO, MgO, Na_2_O, and K_2_O) [23]. However, the OM and PBS used in this study had a low silicon and aluminum content and high Fe content, it was difficult to prepare sintering-expanded haydites. Therefore, through many experiments, we determined that the proportion of raw material should be RBS 20%, OM 10%, PBS 20%, and FA 50%, by changing the SiC foaming agent to prepare the lightweight ceramsite.

**Figure 2:**
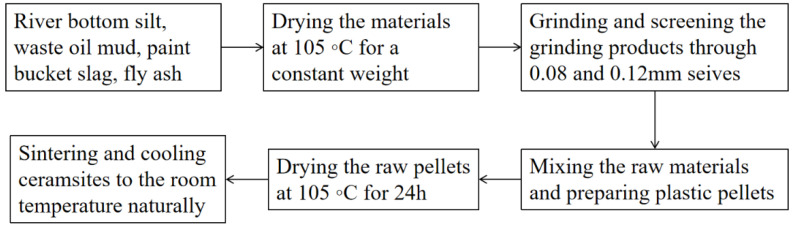
Flowchart of the preparation of solid waste ceramsite.

The preparation process is shown in Figure 2. The detailed preparation process is as follows: RBS, OM, PBS, and FA were dried at 105 °C for 24 h to remove moisture, and then ground in a planetary mill for 1 h. The ground products were passed through an 80-μm sieve and collected for further experiments. According to the mixing proportion, the foaming agent and different dosages of 20% RBS, 10% OM, 20% PBS, and 50% FA were first blended evenly. Prior to the pelletizing process, about 10% water was added. The wet mixture was then handmade into balls of about 8–15 mm in diameter. The raw meal balls were put into a drying oven at 105 °C for 12 h before sintering. Until it reached 600 °C, the sintering rate was 5 °C/min, and then it rose to the highest sintering temperature at a rate of 10 °C/min, and was held for 30 min. When the roasting process was done, the ceramsite was cooled to room temperature in the furnace.

### 2.3. Measurement of Ceramsite Properties

The apparent density, bulk density, 1 h water absorption, and compressive strength of the prepared ceramsites were tested according to the GB/T17431.2-2010 standard of China. The apparent density, bulk density, water absorption, and single particle compressive strength of the samples were calculated by Equations (1)–(4), respectively. To ensure statistical reliability, each sample was tested three times:

(1) $$\rho_{a}=\frac{m_{1}\times 1000}{V_{1}-V_{0}-500}.$$

Here, *ρ_a_* is the apparent density (kg/m^3^), *m*_1_ is the mass of the dried sample (g), *V*_1_ is the total volume of the sample, circular metal plate, and water (mL), and *V*_0_ is the volume of the circular metal plate (mL). The measurement method is as follows: Weigh the dry sample mass, then pour the sample into a 1000-mL measuring cylinder, and then fill it with 500 mL of water. If the sample is floating on the water, press it into the water with a circular metal plate of known volume (*V*_0_) and read the water level of the cylinder (*V*_1_). In this case, the actual volume of the sample is subtracted from the volume of the metal plate (*V*_0_) and 500 mL of water. Calculate the sample apparent density according to Equation (1):

(2) $$\rho_{b}=\frac{\left(m_{2}-m_{3}\right)}{V_{2}}\times 1000.$$

Here, *ρ_b_* is the bulk density (kg/m^3^), *m*_2_ is the total mass of the sample and measuring cylinder (g), *m*_3_ is the mass of the measuring cylinder (g), and *V*_2_ is the volume of the measuring cylinder (mL).

(3) $$\omega_{a}=\frac{m_{4}-m_{5}}{m_{5}}\times 100.$$

Here, *ω_a_* is the water absorption of ceramsite for 1 h (%), *m*_4_ is the mass of the sample after water absorption (g), and *m*_5_ is the mass of the sample before absorption (g). 

However, manual pelletizing can not meet the requirement of the cylindrical compressive strength test, and a large amount of ceramsites is needed. Therefore, a KQ-3 intelligent particle compression machine is used to test the failure load of a single ceramsite. The failure load of the ceramsite is the maximum pressure at which the ceramsite breaks [24,25]. Thirty samples were measured, and the average of these measurements was taken to calculate the compressive strength of the ceramsite by Equation (4):

(4) $$S=\frac{2.8\times PC}{\pi X^{2}}$$

where *S* is the compressive strength of a single ceramsite (MPa), *X* is the distance between the top and bottom indenter (for a spherical ceramsite, the distance is the diameter of the ceramsite (mm)), and *PC* is the failure load of the ceramsite (kN).

### 2.4. Characterization

The thermal behaviors of samples were examined by thermogravimetric analyses and differential scanning calorimetry (TG-DSC) using a ZRY-2P simultaneous TG-DSC analyzer (METTLER TOLEDO, Switzerland), with the samples at a rate of 5 °C/min to 950 °C and then at 10 °C/min to 1200 °C in static air. Major elements of raw materials were analyzed using a X-ray fluorescence spectrometer (S4PIONEER, German Bruker Company, Karlsruhe, Germany). Powder XRD patterns of ceramsite were recorded on a X-ray diffractometer(D/MAX-2200, Japan Rigaku Corporation, Akishima City, Tokyo, Japan) of 50 mA and 40 kV, Cu Kα radiation. SEM-EDS analyses were conducted using the FEI Quanta 200 (FEI Limited, Hillsboro, OR, USA). Ceramsites prepared under different sintering temperatures were ground in a small agate mortar and analyzed by FTIR (Thermo Scientific Nicolet iS5, Waltham, MA, USA), in the wave number range of approximately 400–4000 cm^−1^. In this work, we determined the leaching toxicity of the raw materials and ceramsite prepared under optimal synthetic conditions. The toxic leaching test of the raw material and ceramsite were conducted according to the GB 5085.3-2007 standard of China. Using horizontal oscillation-prepared ceramsite, the extract of the sample with water as the extraction agent, to simulate the leaching of ceramsite by surface water or ground water and the process of harmful components entering the environment. The standard rules on the leaching toxicity identification standard, include inorganic elements and compounds, organic pesticide, non-volatile organic compounds, and volatile organic compounds. Considering that there are almost no organic compounds and volatile substances in ceramsite, heavy metals are mainly detected. The standard includes ICP-AES and ICP-MS for the determination of heavy metals in leaching solution. The concentrations of metal ions were measured by ICP-MS at least three times for each sample in order to ensure statistical reliability.

## 3. Results and Discussion

### 3.1. Effect of SiC Content on Ceramsite Properties

SiC content determines the expansion size, and thus affects the properties of the ceramsite. Therefore, in order to determine the optimum SiC content, a raw material ratio of 20% RBS, 10% OM, 20% PBS, and FA 50% was used for sintering at 1180 °C for 20 min with different SiC dopings. The results are presented in Table 2.

**Figure 3:**
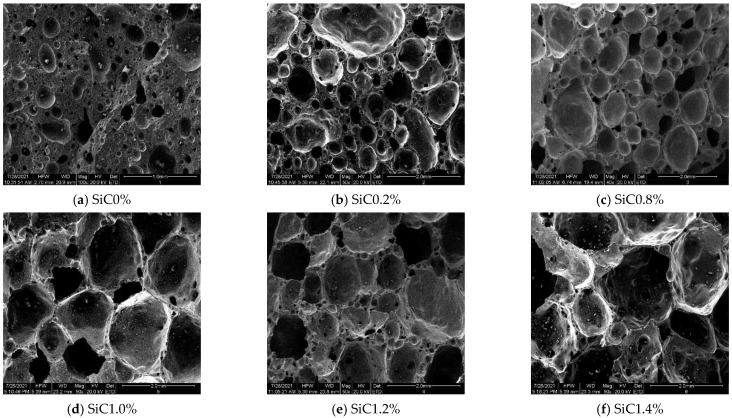
Scanning photomicrographs of internal pores in ceramsite with different SiC contents: (**a**) 0%, (**b**) 0.2%, (**c**) 0.8%, (**d**) 1.0%, (**e**) 1.2%, (**f**) 1.4%.

It can be seen from Table 2 that the porosity of ceramsite was 0.52%, the bulk density was 1870 kg·m^−3^, water absorption was 2.01%, and the single particle compressive strength was 3.98 MPa when SiC is not added, and its bulk density was far from light. With the addition of SiC, the porosity and water absorption of ceramsite gradually increased, while the bulk density and compressive strength showed the opposite trend. This was due to the fact that in the process of preparing ceramsite, SiC produces carbon oxides through its own oxidation, and the sources of oxygen are the air outside the billet and oxygen atoms in the high temperature melt [26]. The oxidation of SiC is intense at about 880 °C, and pores appear obviously at about 1100 °C. With the extension of time, the pores will gradually grow and penetrate [27]. The microstructure of the ceramsite sintered at 1180 °C with different SiC dopings was scanned by electron microscopy, and the results are shown in Figure 3.

It can be clearly seen from Figure 3 that there is a matrix containing a large number of isolated, approximately spherical pores. The formation of these pores is the result of the oxidation of SiC. During the sintering process, gas expansion forms extremely large pores, in which the gas is trapped by the liquid phase. The pores form as the residual glassy phase viscosity falls to a level where gas-forming inorganic decomposition reactions can produce the observed pores [28]. The pore volume in the sintered body increases with the increase of SiC doping, which increases the porosity of the ceramsite. The water absorption of ceramsite is mainly caused by the pore structure on the surface and inside. The internal porosity of ceramsite increases, the structure of ceramsite becomes loose, and then the water absorption rate increases. The large number of large pores contributes to a significant decrease in the compressive strength of ceramsite. In addition, the pores reduce the cross-sectional area of the ceramsite under load. It can be seen from Table 2 that the single particle compressive strength of ceramsite decreased from 3.98 to 0.98 MPa when the porosity of ceramsite increased from 0.52% to 67.95%. With the increase of the number of pores, the compressive strength of ceramsite gradually decreased. At the same time, the micropore structure also concentrates the internal force per unit section and decreases the elastic modulus, which also caused the decrease in the compressive strength of a single particle of ceramsite. Combined with the data in Table 2, the SiC increased from 0.6% to 0.8%, and there was a transient increase in water absorption. With its microscopic morphology, it was speculated that due to the large amount of gas escaping at this time, the melt wrapped with gas covered each other due to the influence of surface tension, forming larger pores. This resulted in significant differences in pore size, so that when the ceramsite was stressed the stress could not be concentrated, the compressive strength dropped steeply, and the water absorption rate increased. When SiC was 1.0%, the microstructure of ceramsite had more uniformly distributed small pores (0.5 mm < pore size < 1.0 mm), interconnected with quartz and other crystals. At this time, the apparent porosity of ceramsite was 23.85% and the bulk density was 490 kg·m^−3^, so SiC of 1.0% was considered to be the best content for the preparation of this lightweight ceramsite.

The main raw materials of the ceramsite were river bottom silt, waste oil mud, paint bucket slag, and fly ash, which contain alkaline substances, heavy metals and other hazardous substances. If the leaching concentration of harmful substances exceeds the regulatory standard, ceramsite production will bring great hazard to natural environment and human health [29]. Hence, the amount of hazardous substances leaching from ceramsites needs to be strictly controlled. The leaching concentrations from C6 of the heavy metals are shown in Table 3. The concentration of all tested heavy metals was lower than the maximum allowable concentration of the leaching solution specified in the national standard (GB 5085.3-2007, Tl no rules), indicating that the prepared solid waste lightweight ceramsite has good stability. The heavy metals were solidified, and this material is not a hazardous waste. We use the SiC as a foaming agent of solid waste ceramsite, not just to deal with a large number of wastes, and it does not pollute the environment. In recent years, lightweight concrete has been used in the field of building materials and the construction industry to achieve energy saving and consumption reduction [30]. This lightweight ceramsite can be used as artificial aggregate to replace natural aggregate to produce lightweight concrete and realize resource utilization [31,32].

### 3.2. Effect of Sintering Temperature on the Sintering of Ceramsite

The properties of ceramsite are greatly affected by sintering conditions, especially sintering temperature. A too low temperature results in an insufficient liquid phase to make ceramsite, while a too high temperature will result in too much liquid phase and damage the structure of the ceramsite. Thus, the raw pellets were sintered at 1140 °C, 1150 °C, 1160 °C, 1170 °C, 1180 °C, and 1190 °C to investigate the effect of temperature on the morphology and properties of the ceramsite.

**Figure 4:**
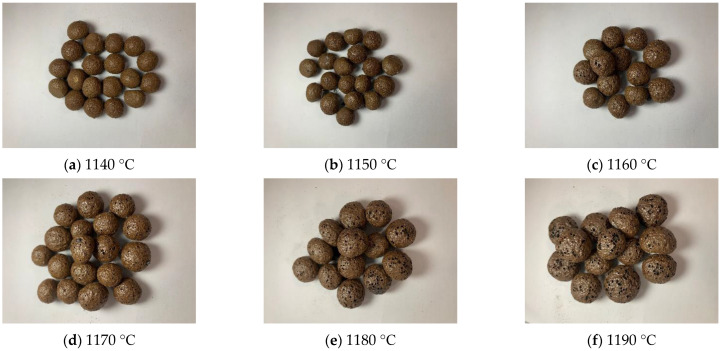
Appearance of ceramsites sintered at different sintering temperatures: (**a**) 1140 °C, (**b**) 1150 °C, (**c**) 1160 °C, (**d**) 1170 °C, (**e**) 1180 °C, (**f**) 1190 °C.

**Figure 5:**
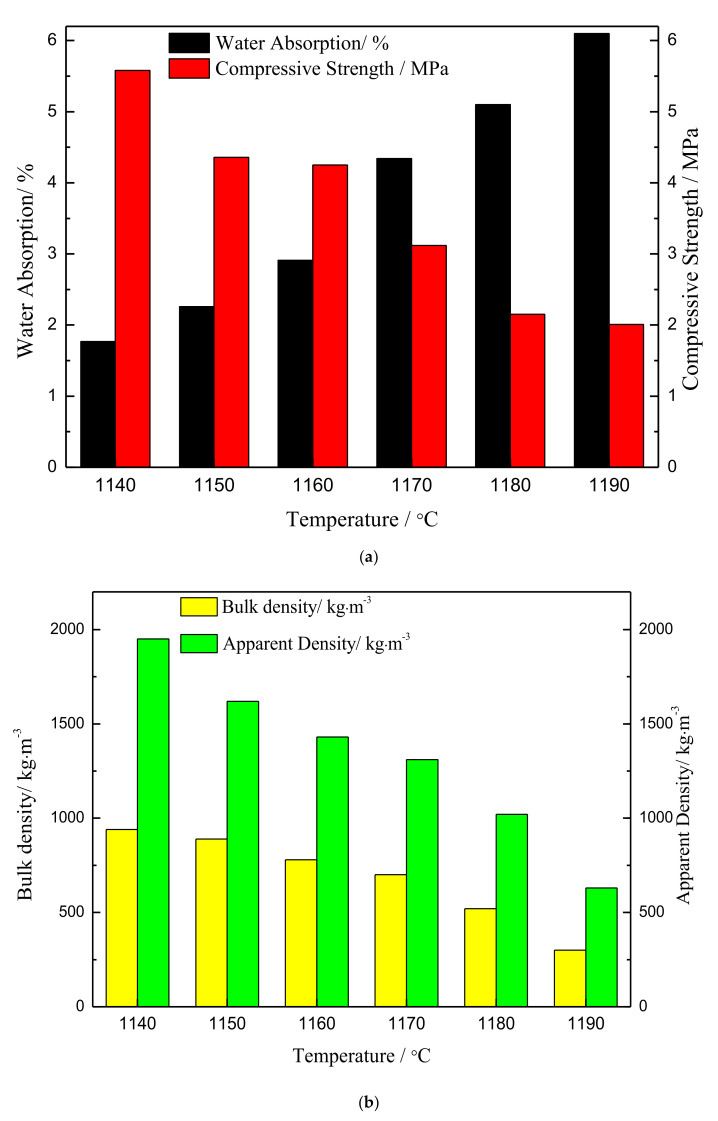
Influence of sintering temperature on ceramsite properties: (**a**) Water absorption and compressive strength; (**b**) bulk density and apparent density.

The appearance and properties of the ceramsites sintered at different temperatures are shown in Figure 4 and Figure 5. At the sintering temperature of 1140 °C, the ceramsite surface was brown and had a dense enamel shell, which reflected that little liquid phase sintering occurred. As the temperature increased further, the surface gradually began to produce a glaze layer with a brown surface and a black interior due to the presence of the iron phase. Moreover, the water absorption increased from 1.77% to 6.10%; the compressive strength decreased from 5.58 to 2.01 MPa; the bulk density and apparent density decreased, and the ceramsite were relatively lightweight. The compressive strength, water absorption, bulk density, and apparent density of ceramsite sintered at 1180 °C were 2.15 MPa, 5.1%, 520 kg/m^3^, and 1020 kg/m^3^, respectively. Observing the ceramsite sintered at 1150 °C, it could be seen that the softening of the balls results in a change of the viscosity and surface tension of the liquid phase, at which time the gas in the particles eventually reaches a dynamic pressure equilibrium. Finally, the pore structure of ceramsite is formed and the strength of ceramsite is decreased [33,34]. More and more pores were created on the surface of the ceramsite due to the oxidation reaction of the SiC with increasing temperature. When the sintering temperature was 1190 °C, collapse of the spherical shape of the ceramsite occurred and large non-uniform pores were observed on the surface. This was mainly due to oversintering, indicating the formation of a large amount of lower viscosity liquid phases during sintering [35]. This formed an excessive amorphous phase, which caused great damage to the structure of the ceramsite. Lightweight ceramsite requires high porosity and low bulk weight, as well as suitable compressive strength, and it is generally considered that the ceramsite with a bulk density of 500 kg/m^3^ belongs to a lightweight aggregate in the ceramsite industry. In actual production, the higher the temperature, the higher the corresponding energy consumption and cost. As can be seen from Figure 4 and Figure 5, the pore distribution and size of ceramsite sintered at 1180 °C are relatively uniform. According to the above analysis, the optimum sintering temperature was selected as 1180 °C.

**Figure 6:**
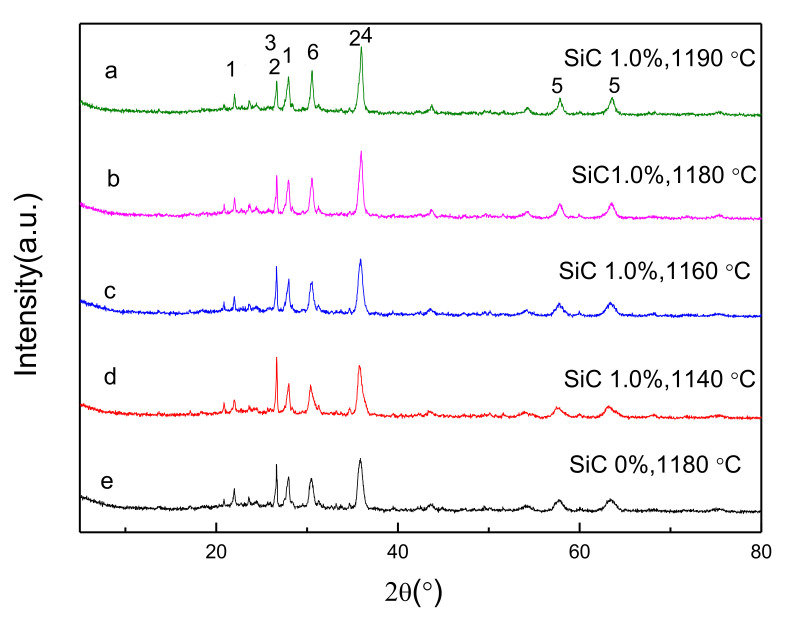
The XRD patterns of ceramsite at different temperatures [1, Albite-low-Na(AlSi_3_O_8_); 2, Kyanite-Al_2_SiO_5_; 3, Quartz-SiO_2_; 4, Fayalite-Fe_2_(SiO_4_); 5, Hematite -Fe_2_O_3_; 6, wurtzute-ZnS].

XRD analyses were applied to obtain mineral compositions of powder ceramsite specimens with different sintering temperatures by using an XRD pattern database (International Centre for Diffraction Data, ICDD). Figure 6 shows that the mineralogical compositions of the ceramsite were very complex due to complex reaction processes, like vaporization, melting, crystallization, bloating, and shrinkage, occurring during the sintering process of the raw materials. The major mineralogical compositions found in the ceramsite consisted of quartz [SiO_2_], fayalite [Fe_2_(SiO_4_)], and kyanite [Al_2_SiO_5_], with small amounts of albite-low [Na(AlSi_3_O_8_)] at different sintering temperatures. Hematite [Fe_2_O_3_], as one of the minor crystalline phases, co-existed with quartz and kyanite. In addition, metallic zinc formed by wurtzute was stabilized. The changes in sintering temperature had little effect on the formation of crystals, and the major crystalline phases remained almost unchanged, as shown in Figure 6. As the sintering temperature increased, the intensity of XRD peaks of Fayalite and kyanite increased, while the quartz phases had a tendency to decrease. As the sintering temperature increased from 1140 °C to 1180 °C, the quartz diffraction peak intensity decreased from 870 CPS to 432 CPS, and the fayalite intensity increased from 754 CPS to 1038 CPS. As the total amount of quartz decreased, an increasing amount of fayalite and kyanite were formed. At 1190 °C, there was still a residual of quartz in the ceramsite, as shown in Figure 6. Comparing Figure 6 (b,e), we found that SiC had no effect on the crystalline phase composition of the ceramsite, although there was a small increase in the content of quartz and its derived minerals [36,37].

**Figure 7:**
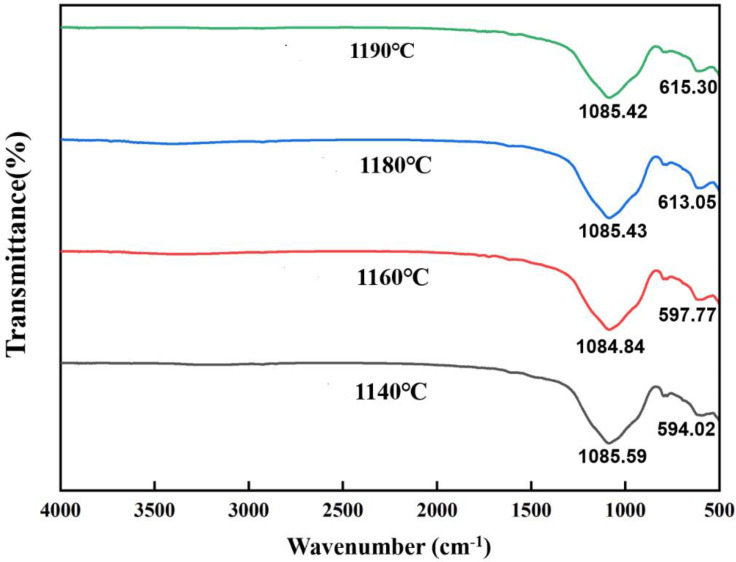
Infrared spectrum of ceramsite sintered at various temperatures.

The results of IR spectroscopy of fired ceramsite at different temperatures (SiC content is 1%) are shown in Figure 7. The analysis showed that the material itself contains a large amount of silica-oxygen tetrahedra (Albite-low, Kyanite, and quartz), which can play a skeletal support role, and its fusion degree affects the burning and swelling process of ceramsite. The fundamental frequency peak around 1085 cm^−1^ had a strong absorbance, and its vibrational frequency was related to the degree of polymerization. The value of 1132 cm^−1^ was the stretching vibrational peak of silica-oxygen tetrahedra (Si-O), and its absorption peak was not affected by the temperature change. The value of 594 cm^−1^–615 cm^−1^ was the characteristic absorption peak of Al(Fe)-O bond in the crystal structure [38,39]. It was obvious that with the increase of the sintering temperature, the wave number of the stretching vibration peak of the Fe-O bond underwent a significant increase [40], which had a large impact on the substance itself. This confirmed the increase of the kyanite and fayalite mineral phases with increasing sintering temperature, as described in the XRD results above.

### 3.3. Sintering Mechanism of Lightweight Ceramsite

**Figure 8:**
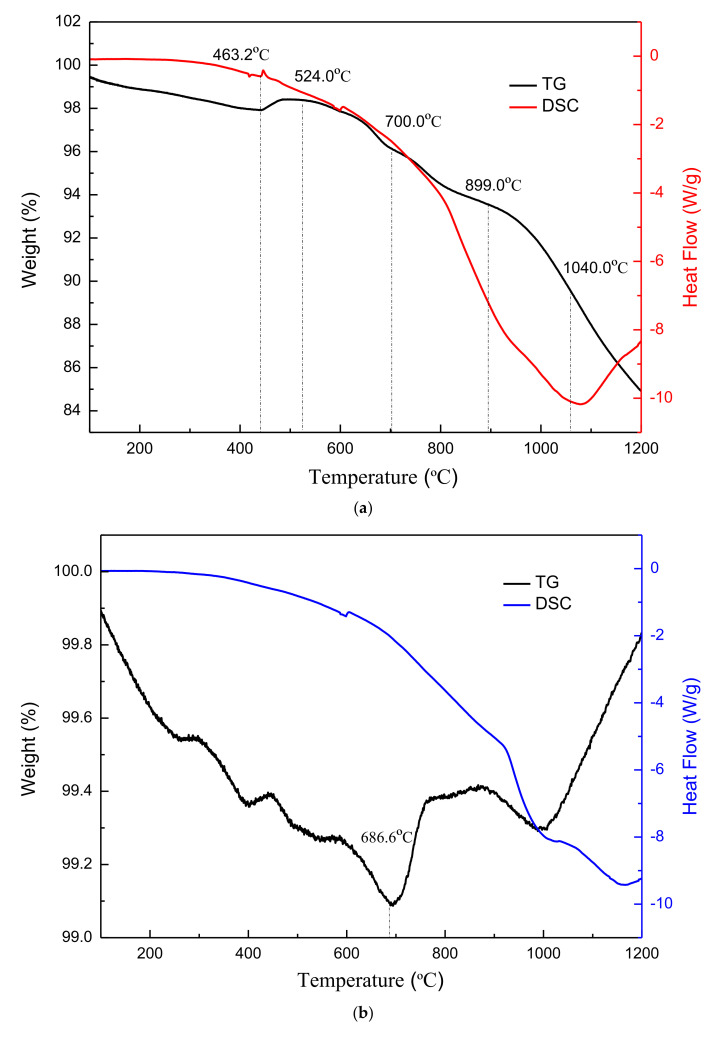

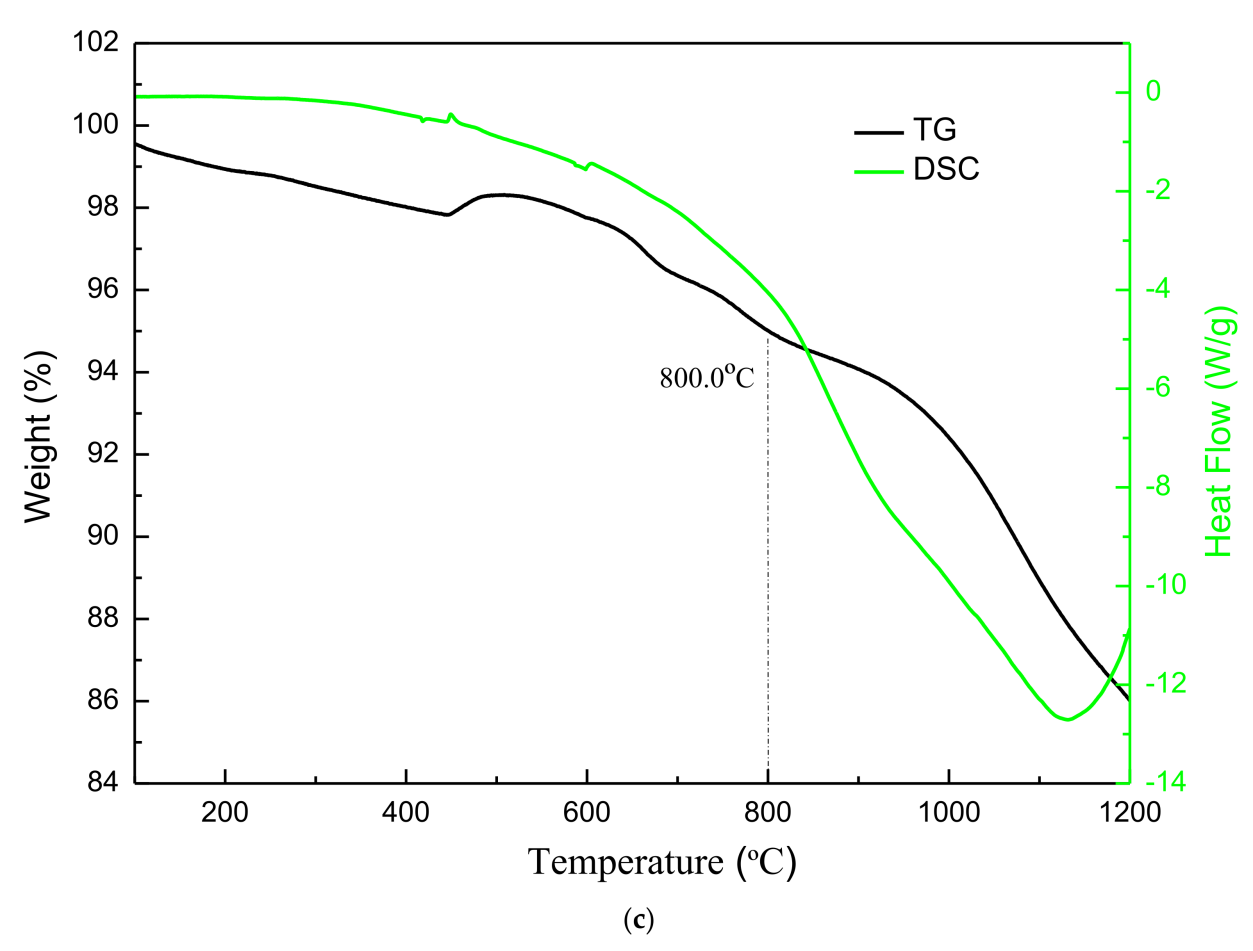
(**a**) TG-DSC analyses of raw material mixtures. (**b**) TG-DSC analyses of SiC. (**c**) TG-DSC analyses of mixtures with 1.0% SiC.

SiC was used as a foaming agent to prepare lightweight ceramic pellets by using its oxidation reaction at high temperatures, releasing gas to form pores inside and on the surface of ceramsite. In order to investigate the process of pore formation, the thermal behaviors of the raw material mixtures (20% RBS, 10% OM, 20% PBS, and FA 50%), SiC alone, and mixtures with 1.0% SiC were examined by TG-DSC. The results are shown in Figure 8.

It can be seen from Figure 8a that the DSC curve of the mixtures does not change much from 50 to 463.2 °C, and shows little weight loss in the TG analysis. An exothermal change was observed in the DSC analysis from 463.2 to 524.0 °C, with 4.9% weight loss, which was caused by the evaporation of water and release of CO_2_. Endothermic change was observed in DSC analysis from 700.0 to 899.0 °C, with a 2.65% weight loss. The weight loss was due to silicate hydrates gradually dehydrating, and it has also been reported that silicate hydrates dehydrate gradually over a wide temperature range up to 800 °C [41]. At about 750 °C, oxide decomposition reactions and conversion of crystalline phases such as quartz occur, and the overall performance is affected by heat absorption [42]. Therefore, the weight loss of raw material mixtures from 700 to 899 °C may be due to the decomposition of the decomposition of oxidede, dehydration of silicate hydrate and crystal transformation of quartz, etc. There are obvious endothermic changes at about 1060.0 °C [28], which indicate the form of the crystalline phase. From the TG curve of Figure 8b, the mass change of SiC with the increase of temperature has two stages: Room temperature ~686.6 °C and 686.6 °C ~ 1200 °C. Room temperature to 686.6 °C is the weight loss stage, and is mainly due to the loss of carbon burning. The oxidation reaction of silicon carbide occurs after about 800 °C and produces a silicon dioxide film covering the surface (SiC + 2O_2_ → SiO_2_ + CO_2_ ↑ [27]), because the relative atomic mass of SiO_2_ is greater than the relative atomic mass of SiC. This is the final embodiment of the weight gain phenomenon [43]. Combining the thermal analysis of the above two substances and comparing Figure 8c, it can be seen that the addition of SiC only had an effect on the rate of weight loss during heating, and the temperature interval of heat absorption and the exotherm hardly moved. Overall, comparing the TG-DSC curves of the mixtures, the addition of SiC did not result in a significant effect on the thermal behavior of the material. However, it is noteworthy that no weight gain occurred for the material in Figure 8c. This is because, with the gradual increase of high temperature silicate melt, the destruction of the silicon dioxide film formed by the oxidation of SiC resulting in the oxidation reaction of silicon carbide has been carried out, and a large amount of gas escapes. The mixed sample in Figure 8c showed a continuous weight loss phenomenon [44].

According to the thermal analysis and physicochemical properties of the above raw materials, it is speculated that the developed microporous structure of ceramsite is mainly caused by the following process: When the raw ceramsite ball is placed in a high temperature environment, the ceramsite is rapidly heated and sintered at a high temperature from outside to inside. The fact that the raw material itself has a lot of C plays a role; the ceramsite outer layer of carbon oxide becomes gas rapidly, while the internal carbon cannot complete oxidation due to the low layer temperature and compactness of the ceramsite material ball. There was only a tiny amount of CO when the ceramsite internal temperature reached around 570 °C, and the internal C with the iron phase from the solid waste in a series of reactions [45]:

3Fe_2_O_3_ + C→2Fe_3_O_4_ + CO_2_↑

Fe_2_O_3_ + 3CO→2Fe + 3CO_2_.

**Figure 9:**
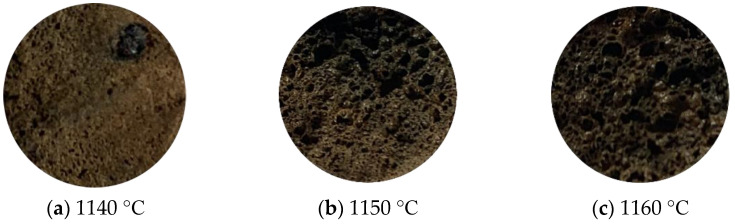

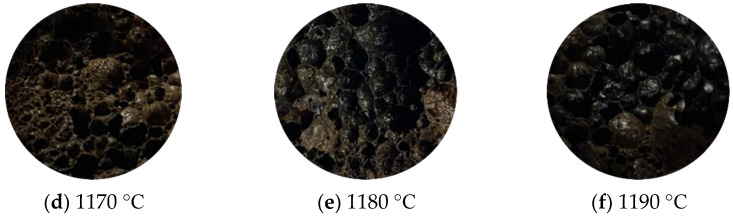
Cross-section of ceramsite at different sintering temperatures: (**a**) 1140 °C, (**b**) 1150 °C, (**c**) 1160 °C, (**d**) 1170 °C, (**e**) 1180 °C, (**f**) 1190 °C.

These reactions are carried out in the process of high temperature sintering of ceramsite. In the process of high temperature sintering of ceramsite, a large amount of expansion gas is generated, so that the volume of the ceramsite expands and a well-developed microporous structure develops without introducing silicon carbide as a foaming agent, as in Figure 4a. After adding SiC, the following reactions occur: SiC + 2O_2_→ SiO_2_ + CO_2_↑, 2SiC + 3O_2_→ 2SiO_2_ + 2CO↑. Oxygen atoms are supplied by oxygen outside the blank or a small amount of oxygen inside. The oxygen inside is due to the fact that at about 1000 °C, hematite will undergo its own decomposition reaction [46,47]: 3Fe_2_O_3_→2FeO + 1/2 O_2_↑, 6Fe_2_O_3_ → 4Fe_3_O_4_ + O_2_↑. When there are alkaline oxides such as MgO and Na_2_O in the system, it will break the Si-O bond and form non-bridging oxygen, which will reduce the degree of polymerization and structural continuity of SiO_2_ and lower the melting temperature of SiO_2_, so that the SiO_2_ film corrodes and the structure becomes loose and its oxidation protection is reduced, allowing oxygen to easily reach the surface of SiC through the SiO_2_ film and the oxidation reaction to continue. The reduced polymerization of SiO_2_ leads to a lower viscosity of the silicate melt, and a large amount of gas is wrapped in the liquid phase of suitable viscosity to form bubbles of different sizes [48]. As the temperature continues to increase, the gas pores undergo the process of growth, fusion, and uplift, and finally a microstructure with obvious gas pores is formed, as shown in Figure 9, when the ceramsite is sintered at 1150–1190 °C. When the liquid viscosity is not appropriate, a large amount of gas will escape, and obvious pores cannot be observed at this time. The internal cross section of ceramsite is still tight, as shown in Figure 9, when the ceramsite is sintered at 1140 °C.

## 4. Conclusions

In this study, river bottom silt, waste oil mud, paint bucket slag, and fly ash were used to prepare lightweight ceramsite, with SiC as the foaming agent. Based on a river bottom silt/waste oil mud/paint bucket slag/fly ash ratio of 2:1:2:5, the effects of SiC doping and sintering temperature on water absorption, bulk density, compressive strength, and apparent density of ceramsite were investigated, and the pore-forming mechanism of ceramsite was also revealed. The following conclusions can be drawn from the experimental results:

1. The dosing of SiC can increase the amount of gas production and thus the formation of pores, causing expansion. The optimum doping amount for the preparation of lightweight ceramsite is 1.0%.
2. The presence of reducing gases causes the redox reaction of hematite to swell the ceramsite and create a microporous structure. The addition of SiC increases the gas production, while the presence of alkaline substances destroys the SiO_2_ film, causing the oxidation reaction to continue, thus making the pellets more porous.
3. The compressive strength, water absorption, bulk density, and apparent density of ceramsite sintered at 1180 °C were 2.15 MPa, 2.1%, 510.3 kg/m^3^, and 1020 kg/m^3^; and the major mineralogical compositions found were quartz [SiO_2_], fayalite [Fe_2_(SiO_4_)], and kyanite [Al_2_SiO_5_], with small amounts of albite-low [Na(AlSi_3_O_8_)].
4. The sintering temperature does not have any effect on the major crystalline phases, which remained almost unchanged. However, the wave number of the stretching vibration peak of the Fe-O bond showed a significant increase with increasing temperature, which is consistent with the pattern of increasing Fe-based mineral content.

## Figures and Tables

**Table 1:** Chemical component analyses of raw materials (wt.%).

	SiO_2_	Al_2_O_3_	Fe_2_O_3_	CaO	K_2_O	MgO	Na_2_O	TiO_2_	SO_3_	P_2_O_5_	ZnO
river bottom silt	63.54	14.58	6.22	5.73	3.85	2.33	1.68	0.87	0.49	0.23	-
waste oil mud	12.36	4.97	52.49	-	1.33	1.67	3.24	-	4.42	1.59	1.51
paint bucket slag	27.18	3.92	18.66	7.48	0.53	1.06	-	1.12	-	4.53	34.21
fly ash	46.09	22.59	8.46	12.12	1.82	1.00	0.78	2.07	4.44	0.24	0.08

**Table 2:** Influence of SiC on properties of ceramsite.

Sample	SiC(wt%)	Porosity/%	Bulk Density/kg·m^−3^	Water Absorption/%	Compressive Strength/MPa
C1	0	0.52	1870	2.01	3.98
C2	0.2	3.26	970	1.99	3.55
C3	0.4	4.12	990	2.32	2.97
C4	0.6	18.72	810	2.48	2.75
C5	0.8	23.50	870	4.52	2.34
C6	1.0	23.85	490	2.02	2.15
C7	1.2	30.86	390	4.81	1.12
C8	1.4	67.95	320	13.16	0.98

**Table 3:** Heavy metal concentration and the national standard.

Heavy Metals/(mg/L)	Ceramsite Leaching Solution/(μg/L)	Limits/(mg/L)
Cd	0.52	1.0
As	56.86	5.0
Hg	0.45	0.1
Tl	<0.3	/
Pb	4.78	5.0

## Data Availability

Data available on request due to restrictions privacy. The data presented in this study are available on request from the corresponding author.
